# Predictive modeling for cow's milk allergy remission by low-dose oral immunotherapy in young children

**DOI:** 10.1016/j.waojou.2024.100910

**Published:** 2024-05-17

**Authors:** Seiko Hirai, Kiwako Yamamoto-Hanada, Kyongsun Pak, Masako Saito-Abe, Tatsuki Fukuie, Yukihiro Ohya

**Affiliations:** a Allergy Center, National Center for Child Health and Development, Tokyo, Japan; b Division of Biostatistics, Clinical Research Center, National Center for Child Health and Development, Tokyo, Japan

**Keywords:** Allergy, Cow's milk, Immunotherapy, Predictive modeling, Remission

## Abstract

**Background:**

The effectiveness of slow low-dose oral immunotherapy (SLOIT) for cow's milk (CM) allergy has been reported. Most OIT studies have discussed the target populations over 4 years old. Furthermore, no predicting modeling is reported for CM allergy remission by CM-SLOIT under 4 years of age.

**Objective:**

We sought to develop a predictive model for CM allergy remission by SLOIT after 3 years in young children who started CM-SLOIT under 4 years of age.

**Methods:**

We included young children with cow's milk allergy or cow's milk sensitization (development modeling set with 120 children and validation modeling set with 71 children). We did logistic regression analysis to develop the models. We calculated the area under the receiver operating curves (ROC-AUCs) to evaluate the predictive modeling performance.

**Results:**

The model (CM-sIgE before SLOIT + age at beginning SLOIT + serum TARC before starting SLOIT + CM-sIgE titer one year after OIT) showed good discrimination with the ROC-AUC of 0.83 (95% CI:0.76–0.91) on internal validation. Applying the model to the validation set gave good discrimination (ROC-AUC = 0.89, 95% CI:0.80–0.97) and a reasonable calibration (intraclass correlation coefficient = 0.88, 95% CI:0.62–0.97).

**Conclusion:**

We developed and validated predictive modeling for determining the remission rate of CM allergy at 3 years after SLOIT under 4 years of age in children with CM allergy. This predictive model is highly accurate and can support CM allergy management. (226 words)

## Introduction

The food allergy epidemic is a global issue that imposes a heavy burden.^1^ Cow's milk allergy is one of the most frequent causal food allergens in food allergy children.^2^^,^^3^ Absolute causal food avoidance delays food allergy tolerance induction.^4^ Furthermore, children of high IgE-sensitized cow's milk could not obtain natural remission with a lifelong burden.^5^ The Japanese food allergy guidelines have not recommended absolute causal food elimination but minimum causal food elimination based on the results of an oral food challenge to confirm the threshold.^6^

A systematic review addressed that oral immunotherapy for cow's milk had highly contributed to food allergy treatment, but adverse allergic reactions were common.^7^ The guideline recommends oral immunotherapy for food allergy starting around 4–5 years old.^8^ Slow low-dose oral immunotherapy is one of the oral immunotherapy methods avoiding serious adverse reactions that proceed below the threshold level by inducing immune tolerance by starting with a small amount of allergen, slowly increasing the intake, and confirming an increase in the threshold level and continuing with partial elimination.^9^^,^^10^ We consider that slow, low-dose oral immunotherapy is also useful in infants. Taking even small amounts of allergenic foods could increase the thresholds and reduce allergic symptoms by accidental causal food intake.

A recent review indicated that oral immunotherapy was initiated in infants at the age of 3 months. There is no global harmonized definition of cow's milk tolerance. However, in Japan, the standardized definition for cow's milk complete remission is availability of 200 ml of cow's milk (6.6 g of cow's milk protein), and we use this definition in school lunch management.^6^ Children who can take 10 ml of cow's milk are usually able to take confectionery containing cow's milk.

There might be multiple factors influencing tolerance induction. Excellent eczema management itself could reduce IgE titers.^11^ Early introduction of allergenic foods could induce tolerance induction. However, affecting factors need to be better-recognized. Caregivers want to know the future prognosis of slow low-dose oral immunotherapy in cow's milk. However, we have not had a predicting model for cow's milk tolerance by slow low-dose oral immunotherapy under four years of age. We sought to develop a predictive model for cow's milk tolerance by slow low-dose oral immunotherapy in the first three years in young children with cow's milk allergy who started under four.

## Methods

### Participants and study design

**Fig. 1:**
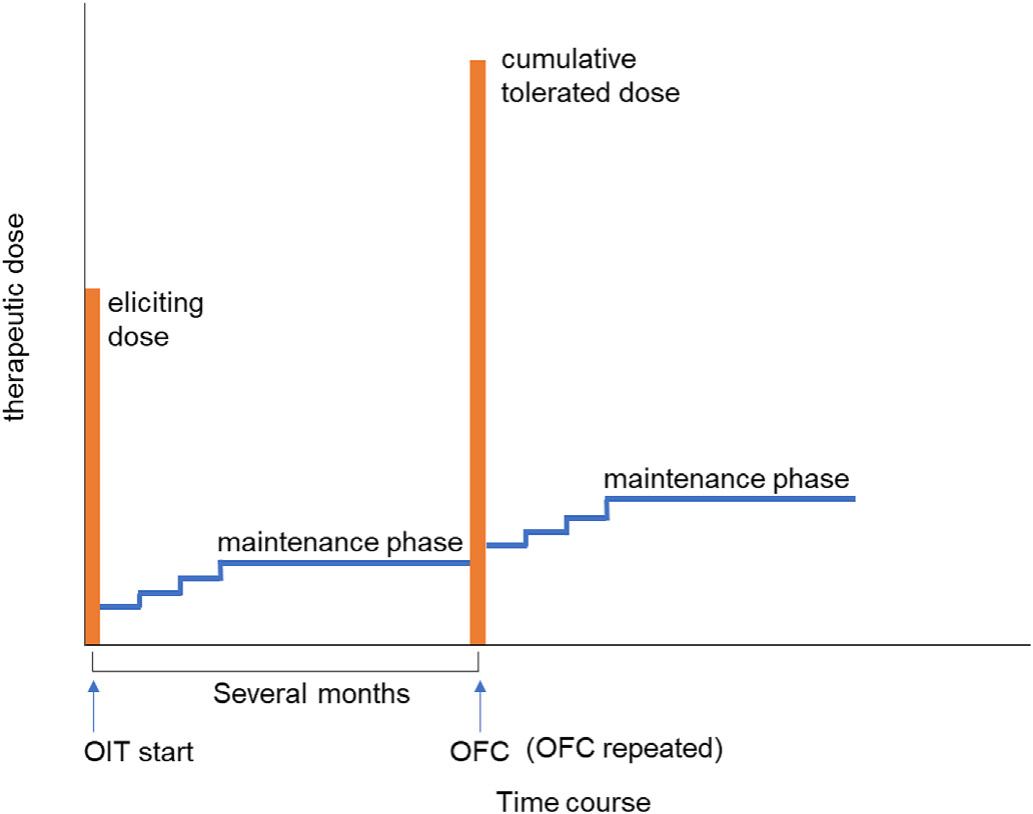
A regimen of slow low-dose oral immunotherapy

We collected medical chart records of the children with cow's milk sensitization or cow's milk allergy. Eligible populations were as follows: 1) the first visit from January 2014 and October 2018, 2) sensitized to cow's milk, 3) complete elimination for cow's milk and dairy products, 4) started slow low-dose oral immunotherapy^9^ with cow’s milk under 4 years of age, and 5) slow low-dose oral immunotherapy followed for 3 years. The slow, low-dose oral immunotherapy protocol details were shown in Fig. 1. Physicians decide the starting and maintenance doses regarding the cumulative tolerated dose or/and eliciting dose based on oral food challenge (OFC) results or previous allergic reactions. In our institution, the starting dose was usually set at 1/10 and lower of the eliciting dose. Whereas conventional oral immunotherapy is defined as continuing ingestion of allergenic food above the threshold, we set the initial and maintenance dose below the threshold for slow low-dose oral immunotherapy. In case children were able to continue taking the maintenance dose for several months, we did OFC to confirm the elevated eliciting or tolerated dose and increased the therapeutic dose slowly to the maintenance dose below the threshold. This procedure was repeated to increase the treatment dose as slow low-dose oral immunotherapy safely. We included cases where oral food challenge was not performed before the start of slow low-dose oral immunotherapy. The initial intake was determined in those children based on past allergic history. The treatment foods were orally administered. Children with slow low-dose oral immunotherapy took non-heated cow's milk or yogurt made with cow's milk (if the children needed to take a tiny amount of treatment food, they could take it as homemade pancakes or steamed bread)^12^ at home.

Treatment foods were determined by children's and caregivers' preferences based on personalized approaches and shared-decision making. Exclusion criteria were as follows: 1) loss to follow-up, 2) slow low-dose oral immunotherapy discontinuation of more than 3 months, and 3) with non-immediate reactions. This study was approved by the institutional ethical review board (2022-057). The development modeling set included 120 children who first visited from January 2014 to December 2016. The validation modeling set included 71 children who first visited from January 2017 to October 2018. We defined the primary outcome as drinking 200 ml of cow's milk (6.6 g of cow's milk protein) in the first 3 years. Secondary outcomes were 10 ml cow's milk (0.33 g of cow's milk protein) intake in years 1, 2, and 3 after slow low-dose oral immunotherapy initiation.

We analyzed the comparison between 2 dataset groups using Student's *t*-test, Welch's *t*-test, or chi-square test. We reported the associations of specific parameters with primary outcomes using odds ratios (ORs) with 95% confidence intervals (CIs). A P value of <0.05 was defined to indicate statistical significance. All statistical analyses were performed with EZR and R version 4.1.3 (The R Foundation for Statistical Computing, Vienna, Austria) for predictive modeling.^13^

We selected variables for developing predictive models from previously published reports and clinically important items. Models were as follows: Model 1: cow's milk-sIgE before slow low-dose oral immunotherapy + age at beginning oral immunotherapy; Model 2: Model 1 + TARC (thymus and activation-regulated chemokine),^14^ serum biomarker of atopic dermatitis severity (commercially available in Japan), before slow low-dose oral immunotherapy initiation; and Model 3: Model 2 + cow's milk -sIgE 1 year after slow low-dose oral immunotherapy. We created the models by logistic regression analysis using the development modeling dataset. A 3-fold cross-validation was performed 100 times for internal validation and bootstrapped 2000 times to construct 95% confidence intervals for the receiver operating characteristic curve (ROC)- The Area Under the Curve (AUC). External validation was performed using calibration plots based on the validation modeling dataset.

### Variable definitions

We used the capsulated hydrophilic carrier polymer (CAP) method to measure food antigen-specific antibody titers. This test method indicates a titer of 100 UA/ml or higher as “≥100 UA/ml”. All≥100 UA/ml were analyzed for statistical analysis as 100 UA/ml. Severe atopic dermatitis was defined as EASI score >21.1 or SCORAD index >50. The grade of allergic symptoms was based on the severity classification of the anaphylaxis guidelines.

### Handling of missing values

We did multiple imputations to perform completion on missing data and analyze with the maximum number of cases. Four Variables were used in multiple imputations: gender, presence of atopic dermatitis at first visit, total IgE level before the start of slow low-dose oral immunotherapy, and cow's milk-sIgE level before the start of slow low-dose oral immunotherapy. A biostatistician supported statistical analysis.

## Results

**Fig. 2:**
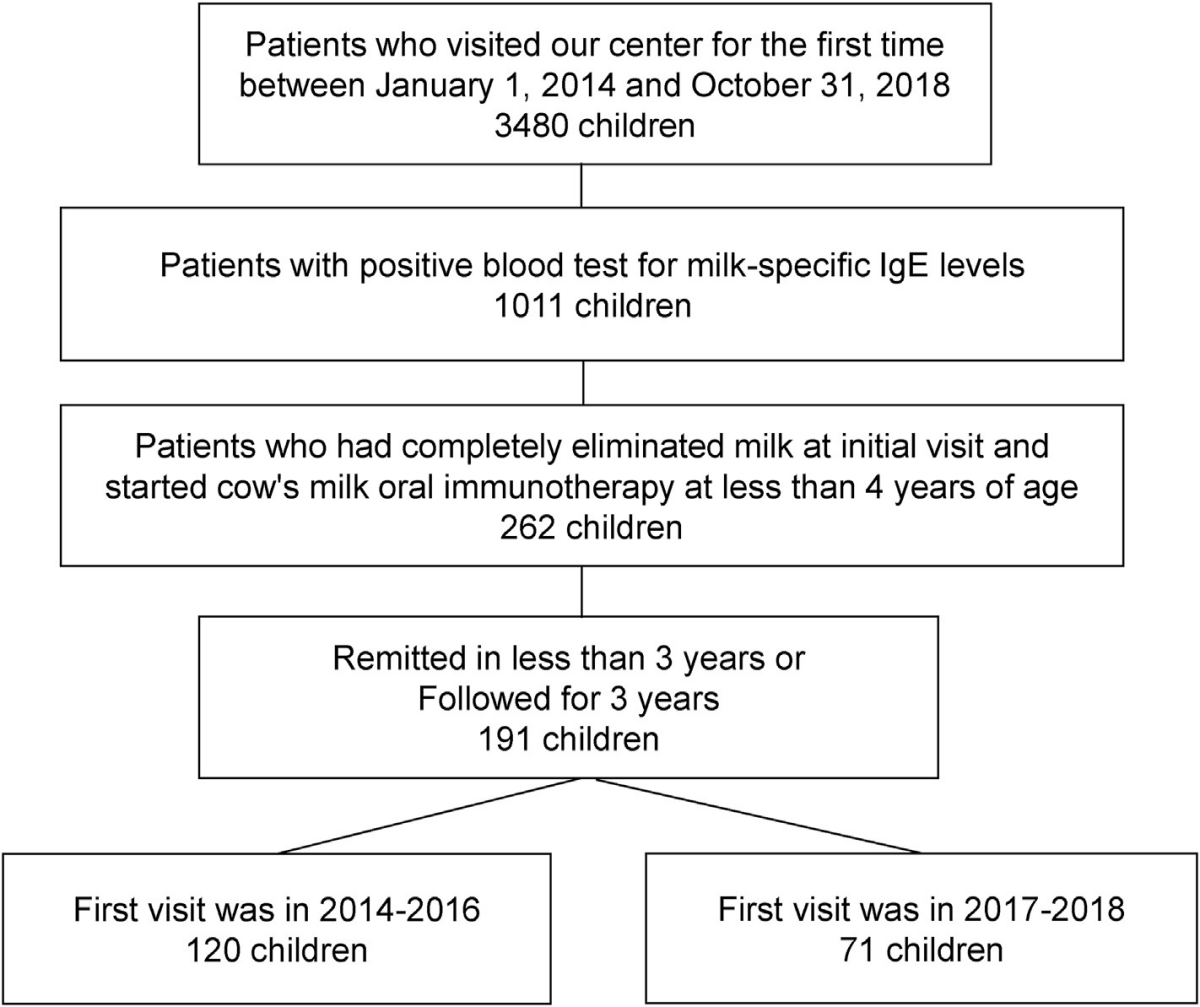
Flow chart of the study

Of the 3480 children who visited the allergy center for the first time during the relevant period, 1011 were sensitized to cow's milk-sIgE (see Fig. 2). Of these, 262 children had eliminated dairy products at their first visit and had started slow low-dose oral immunotherapy for cow's milk under 4 years of age. A total of 191 (=120 + 71) children obtained cow's milk remission within 3 years or could be followed for 3 years. The clinical characteristics of the children are shown in Table 1. 176 children (92.1%) had atopic dermatitis at the first visit. Table 2 demonstrates slow low-dose oral immunotherapy information. The median age in months at slow low-dose oral immunotherapy start was 19 months. The mean slow low-dose oral immunotherapy starting amount of cow's milk was 1.6 ml. On average, the children drank 28.8 ± 54.6 ml after 1 year, 69.3 ± 85.2 ml after 2 years, and 93.4 ± 93.0 ml after 3 years. After 3 years of slow low-dose oral immunotherapy, 61% of children could take 10 ml of cow's milk (0.33 g of cow's milk protein), and 40% could drink 200 ml of cow's milk (6.6 g of cow's milk protein). During slow low-dose oral immunotherapy, anaphylaxis was only observed 1 (0.83%) in the development dataset and 2 (2.8) % in the validation dataset. However, anaphylaxis related to accidental cow's milk intake, except slow low-dose oral immunotherapy, was more frequent than anaphylaxis events related to slow low-dose oral immunotherapy (see Table S1). Table S2 demonstrates the results of blood tests. Cow's milk-sIgE, before slow low-dose oral immunotherapy started, was 30.1 ± 33.8 UA/mL (mean ± SD).

**Table 1:** Characteristics of the children in this study.

Variable		Development set	N	Validation set	N	P value
Sex						
Boy	(%)	78 (65.0)	120	51 (71.8)	71	0.343
Atopic diseases^a^						
AD	(%)	113 (94.2)	120	63 (88.7)	71	0.265
Severe AD^b^	(%)	17 (18.9)	90	3 (7.0)	43	0.117
EASI>21.1	(%)	8 (14.3)	56	2 (5.0)	40	0.114
SCORAD>50	(%)	9 (26.5)	34	1 (33.3)	3	1.0
Asthma	(%)	18 (15.0)	120	11 (15.5)	71	1.0
Allergic rhinitis	(%)	0 (0)	120	0 (0)	71	–
FA other than CM	(%)	107 (89.2)	120	64 (90.1)	71	1.0
OIT other than CM	(%)	108 (90.0)	120	63 (88.7)	71	0.81
Siblings^a^	(%)	50 (43.1)	116	32 (45.1)	71	0.879
Family's history^a^						
Family's allergic history	(%)	111 (95.7)	116	65 (91.5)	71	0.338
Parent's history of FA	(%)	31 (26.7)	116	21 (29.6)	71	0.737
Siblings' history of FA	(%)	19 (16.4)	116	13 (18.3)	71	0.842
OFC before OIT	(%)	95 (79.2)	120	48 (67.6%)	71	0.0858
Judgment						0.0139
Negative	(%)	17 (17.9)	95	15 (31.3)	48	
Positive	(%)	72 (75.8)	95	28 (58.3)	48	
OAS only	(%)	2 (2.1)	95	5 (10.4)	48	
difficult to judge	(%)	4 (4.2)	95	0 (0)	48	
Reactive amount in OFC (mean, SD)	g	11.2 ± 20.5	95	10.7 ± 19.2	48	0.894
Reactive amount with <10 g	(%)	56 (75.7)	74	20 (58.8)	34	0.111
Cumulative amount in OFC (mean, SD)	g	14.5 ± 34.8	95	6.9 ± 13.2	48	0.0851
Anaphylaxis	(%)	25 (26.3)	95	8 (16.7)	48	0.215
Use of epinephrine	(%)	0 (0)	95	2 (4.2)	48	0.111
History of immediateAllergic reactions before OIT	(%)	104 (86.7)	120	55 (77.5)	71	0.112
History of Anaphylaxis before OIT	(%)	41 (34.2)	120	18 (25.4)	71	0.257

AD: atopic dermatitis, FA: food allergy, CM: cow's milk, OIT: oral immunotherapy, OFC: oral food challenge, OAS: oral allergy syndrome, SD: standard deviation.

a Information at the time of the initial visit.

b Those that meet EASI>21.1 or SCORAD>50

**Table 2:** Outcomes by OIT.

Variable		Development set	N	Validation set	N	P value
Age at beginning OIT (mean, SD)	month	22.5 ± 10.1	120	21.5 ± 13.2	71	0.572
Amount of starting OIT (mean, SD)	g	2.2 ± 7.2	120	0.7 ± 1.2	71	0.0238
Amount in the 1st 1 year of OIT (mean, SD)	ml	34.9 ± 62.9	120	18.4 ± 34.8	71	0.0442
Amount in the 1st 2 year of OIT (mean, SD)	ml	69.9 ± 85.9	120	68.3 ± 84.6	71	0.899
Amount in the 1st 3 year of OIT (mean, SD)	ml	92.8 ± 93.1	120	94.5 ± 93.4	71	0.905
Reached 10 ml in the 1st 1 year of OIT	(%)	43 (35.8)	120	25 (35.2)	71	1.0
Reached 10 ml in the 1st 2 year of OIT	(%)	61 (50.8)	120	36 (50.7)	71	1.0
Reached 10 ml in the 1st 3 year of OIT	(%)	73 (60.8)	120	44 (62.0)	71	1.0
Reached 200 ml in the 1st 1 year of OIT	(%)	12 (10.0)	120	1 (1.4)	71	0.0335
Reached 200 ml in the 1st 2 year of OIT	(%)	33 (27.5)	120	18 (25.4)	71	0.866
Reached 200 ml in the 1st 3 year of OIT	(%)	48 (40.0)	120	29 (40.8)	71	1.0
Months reaching 10 ml (mean, SD)	month	38.4 ± 21.3	84	34.4 ± 21.7	48	0.303
Months of remission (mean, SD)	month	47.6 ± 20.3	60	43.8 ± 16.1	36	0.331
Form of OIT^a^			120		71	0.758
Milk	(%)	94 (78.3)		51 (71.8)		
Yogurt	(%)	11 (9.2)		9 (12.7)		
Commercial processed products	(%)	5 (4.2)		3 (4.2)		
Handmade pancake	(%)	10 (8.3)		8 (11.3)		
Allergic reaction	(%)	44 (36.7)	120	25 (35.2)	71	0.963
Maximum Grade			44		25	0.37
Grade 1	(%)	41 (93.2)		23 (92.0)		
Grade 2	(%)	2 (4.5)		0 (0)		
Grade 3	(%)	1 (2.3)		2 (8.0)		
Symptomatic organs			44		25	
Only Skin	(%)	29 (65.9)		18 (72.0)		0.863
Respiratory symptoms	(%)	11 (25.0)		6 (24.0)		1.0
Other than respiratory	(%)	4 (9.1)		1 (4.0)		0.653
Anaphylaxis	(%)	1 (0.83)	120	2 (2.8)		0.643
Use of epinephrine	(%)	0 (0)	120	1 (1.4)		0.79
Emergency room visit	(%)	4 (3.3)	120	3 (4.2)		1.0

OIT: oral immunotherapy, SD: standard deviation, AR: allergic reaction, AI: accidental ingestion.

a If not one, the main one used

Table 3, Table 4 and Table S3 show the internal validation results with the adjusted model obtained from the development modeling set in Supplemental Figs. 3 and 4, demonstrating the results of calibration plots. For the primary outcome (200 ml cow's milk [6.6 g of cow's milk protein] intake 3 years later), Model 1 demonstrated AUC 0.80 (95%CI: 0.72–0.88), Model 2 showed AUC 0.80 (95%CI: 0.72–0.88), Model 3 demonstrated AUC 0.83 (95%CI: 0.76–0.91).

**Table 3:** Multivariable associations between the predictors and the primary outcome in the development set (Model 3).

Intercept and predictors	aOR	95%CI
Intercept	2.018	0.383, 10.625
CM-sIgE before OIT initiation	0.995	0.955, 1.036
Age at beginning OIT	0.958	0.902, 1.018
TARC before OIT initiation	1	1, 1.001
CM-sIgE 1 year after OIT	0.925	0.864, 0.992

CM: cow's milk, OIT: oral immunotherapy, OR: Odds ratio, CI: confidence interval

**Table 4:** Multivariable associations between the predictors and the secondary outcome (drink 10 ml after 1, 2 or 3 year) in the development set (Model 3).

Intercept and predictors	1 year	2 years	3 years
	**aOR(95%CI)**		
Intercept	1.736 (0.323, 9.348)	3.389 (0.541, 21.24)	4.858 (0.756, 31.235)
CM-sIgE before OIT initiation	0.986 (0.939, 1.035)	0.991 (0.95, 1.033)	1.01 (0.979–1.042)
Age at beginning OIT	0.965 (0.907, 1.026)	0.966 (0.91, 1.026)	0.971 (0.916, 1.029)
TARC before OIT initiation	1.001 (1, 1.001)	1.001 (1, 1.002)	1.001 (1, 1.001)
CM-sIgE 1 year after OIT	0.916 (0.845, 0.993)	0.904 (0.842, 0.971)	0.928 (0.888, 0.97)

CM: cow's milk, OIT: oral immunotherapy, OR: Odds ratio, CI: confidence interval

**Fig. 3:**
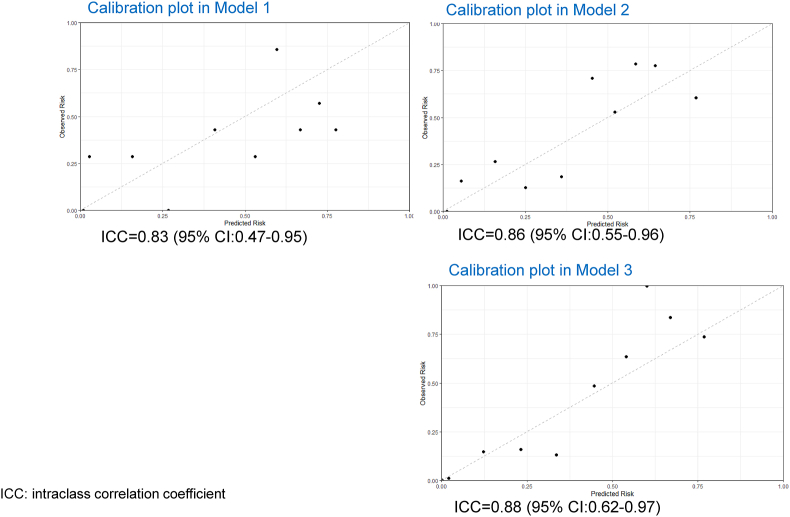
Calibration plots in Models 1, 2 and 3 for the primary outcome. Model-specific calibration plots in the external validation of the predictive model with the primary outcome (200 ml cow's milk intake three years later)

**Fig. 4:**
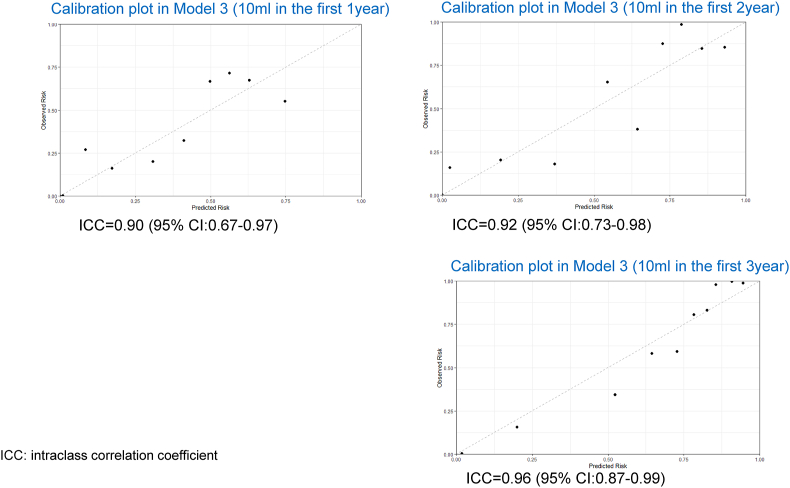
Calibration plots in Models 1, 2 and 3 for the secondary outcomes. Calibration plots for external validation in Model 3 to predict secondary outcomes (drinking 10 ml cow's milk within 1, 2 and 3 years)

For the secondary outcome (10 ml cow's milk [0.33 g of cow's milk protein] after 1, 2, and 3 years), Model 1 demonstrated AUC 0.80 (95%CI: 0.73–0.88) for 10 ml after 1 year, AUC 0.83 (95%CI: 0.76–0.90) for 10 ml cow's milk after 2 years, and AUC 0.81 (95%CI: 0.73–0.89) for 10 ml cow's milk after 3 years. Model 3 showed excellent discrimination with the AUC 0.83 (95% CI:0.76–0.91) on internal validation (see Table S3). Application of the model in the validation set gave an excellent discrimination (AUC 0.89, 95% CI:0.80–0.97) and a reasonable calibration (intraclass correlation coefficient [ICC] = 0.88, 95% CI:0.62–0.97) (see Fig. 3). For secondary outcomes, internal validation and external application in the validation modeling set showed the results in Table S4 and Fig. 4.

## Discussion

We developed and validated predictive modeling for determining the remission rate 3 years after slow low-dose oral immunotherapy under 4 years of age in children with cow's milk allergy. This predictive model is highly accurate and can support cow's milk allergy management. Variables included in the model (Cow's milk-sIgE before slow low-dose oral immunotherapy + age at beginning slow low-dose oral immunotherapy + TARC (eczema severity marker) before slow low-dose oral immunotherapy initiation + cow's milk-sIgE 1 year after slow low-dose oral immunotherapy) were reasonable. Future prognosis is influenced not only by a single factor but multiple factors such as age, sIgE titers, eczema management, Etc. We need to consider various factors that affect the efficacy and future prognosis of oral immunotherapy.

One of the novel points in this study is that we included atopic dermatitis severity (TARC) in our modeling with excellent accuracy. TARC is a type of chemokine produced by epidermal keratinocytes, and serum TARC levels reflect the severity of atopic dermatitis. TARC is correlated with SCORAD score, so a higher serum TARC level indicates that atopic dermatitis is more severe. As we know, several studies demonstrated that food allergy was deeply associated with atopic dermatitis.^15^, ^16^, ^17^ As we mentioned before, improved eczema management itself could reduce IgE titers. In our previous randomized controlled trial (RCT) investigating immune tolerance induction for infants, poorly controlled atopic dermatitis infants they developed hen's egg allergy even though they took a tiny dose of hen's egg. A recent RCT also demonstrated that eczema management via skin intervention could reduce hen's allergy in infants.^18^ Exposures to peanuts in dust and sensitization levels differed among eczema and non-eczema children.^19^ Based on the dual allergen hypothesis, eczema management via skin and oral allergenic intervention is also necessary for tolerance induction in treating food allergy and preventing food allergy.^20^ Various food allergens such as peanuts, hen's egg, and walnuts are in our environment,^19^^,^^21^^,^^22^ so the skin barrier is also crucial to prevent percutaneous IgE sensitization via inflamed skin. Before oral immunotherapy, children need to keep eczema controlled well. Our model could show eczema management's importance in children with atopic dermatitis and food allergy.

As for including the variable of IgE sensitization, our study results coincided with a past study^23^ since Peterson et al demonstrated that the level of sIgE had a greater risk predictive factor for cow's milk remission.^23^ We empirically understand that cow's milk children with a significant decline in cow's milk-sIgE about 1 year after initiation of oral immunotherapy can achieve early remission from clinical experience. We included cow's milk-sIgE one year after initiation of slow low-dose oral immunotherapy as a variable in our model and provided accuracy and fit in the predictive model. We consider that IgE titer trajectory at the one-year point after the start of treatment is valuable to capture the future prognosis of cow's milk allergy by oral immunotherapy.

In consultation with biostatisticians, we selected variables from previously published reports and clinically important items. Because there are multiple factors associated with food allergy prognosis, in this study, we aim to combine several variables to create a model and then add several more variables to make the prediction more accurate. Since we do not have a large sample size, we would like to examine which factors are more influential in the subsequent study using extensive data from future studies. We believe this prediction modeling is helpful before we discuss with children and families their future cow's milk allergy prognosis through slow low-dose oral immunotherapy. Based on this prediction model, we plan to develop a simplified model equation that can be used in clinical practice in coordination with statisticians.

The strength was that slow low-dose oral immunotherapy was safe, and anaphylaxis was rare. Children and families could safely be satisfied with slow low-dose oral immunotherapy introduction without anxiety. However, we have several limitations of this study. First, this was a retrospective study and a single-center study. Now, we have an ongoing prospective observational cohort study for food allergy. Second, the detailed slow low-dose oral immunotherapy regimen was decided based on shared-decision making, but our data reflected a real-world clinical practice. Third, we focus on only cow's milk allergy in this study but would like to develop another predictive model for hen's egg, peanut, and tree nuts allergies. Fourth, although TARC^24^ is approved as a clinical test in Japan, it is not globally available in clinical practice. In this study, because of the large number of missing EASI or SCORAD variables, we chose TARC. Since TARC was highly correlated with SCORAD,^24^ we assumed that the accuracy of the predictive model would be equivalent even if the variables were replaced from TARC to SCORAD. We hope to release the next version with SCORAD as variables for practical use.

## Conclusion

We developed and validated predictive models for determining the remission rate three years after slow low-dose oral immunotherapy initiation at less than four years of age in young children with cow's milk allergy. This model is highly accurate and may help manage cow's milk allergy.

## Abbreviations

AUC, Area Under the Curve; CAP, capsulated hydrophilic carrier polymer; CI, confidence interval; CM, cow's milk; ICC, intraclass correlation coefficient; OR, odds ratios; RCT, randomized controlled trial; SLOIT, Slow Low-dose oral immunotherapy; ROC, receiver operating characteristic curve; TARC, thymus and activation-regulated chemokine

## Acknowledgments

This study was supported by a grant from 10.13039/100007786National Center for Child Health and Development.

## Funding

National Center for Child Health and Development.

## Availability of data and materials

The data that support the findings of this study, which are captured in the current article, are available from the corresponding author upon reasonable request.

## Authors’ information

Seiko Hirai: email; hirai-s@ncchd.go.jp, authors’ consent for publication; obtained, author contributions; conceptualization, methodology, visualization, writing, review, and editing.

Kiwako Yamamoto-Hanada: email; yamamoto-k@ncchd.go.jp, authors’ consent for publication; obtained, author contributions; supervision, writing, review, editing and project administration.

Kyongsun Pak: email; pak-k@ncchd.go.jp, authors’ consent for publication; obtained, author contributions; methodology, validation, formal analysis, investigation and data curation.

Masako Saito-Abe: email; saito-myk@ncchd.go.jp, authors’ consent for publication; obtained, author contributions; conceptualization, methodology and review.

Tatsuki Fukuie: email; fukuie-t@ncchd.go.jp, authors’ consent for publication; obtained, author contributions; conceptualization, methodology and review.

Yukihiro Ohya: email: ohya-y@ncchd.go.jp, authors’ consent for publication; obtained, author contributions; conceptualization, methodology, review and project administration.

## Ethics statement

The study was reviewed and approved by an institutional review board (IRB). All research was conducted in accordance with Declaration of Helsinki and its later amendments.

This study was approved by the IRB of the National Center for Child Health and Development (2022-057).

## Declaration of competing interest

**S Hirai** had no conflict of interest related to this study.

**K Pak** had no conflict of interest related to this study.

**K Yamamoto-Hanada** had a grant from 10.13039/100007786National Center for Child Health and Development (2023B-14) related to this study. She received grants from 10.13039/100009619Japan Agency for Medical Research and Development (Grant Number JP22he0422021j0001), 10.13039/501100001691JSPS KAKENHI (Grant Number: 22K10545, 20H05678, 19H03569, 18H03101, and 17K13214), 10.13039/100007786National Center for Child Health and Development (Grant Number: 2019B-1 and 2021B-12), Japanese Society of Allergology, 10.13039/100014423Environmental Restoration and Conservation Agency, National Science, and Takano Medical, consulting fees from bcase, 10.13039/100006483AbbVie and KAO, and lecture fees from 10.13039/100006483AbbVie, KAO, 10.13039/501100007132Otsuka Pharmaceutical, Torii Pharmaceutical, Maruho, 10.13039/100004319Pfizer, and 10.13039/100004339Sanofi out of submitted work. She is a member of the allergy prevention committee (World Allergy Organization) and a board member of the Japan Society of Health Science for Children out of submitted work.

**M Saito-Abe** had a grant from 10.13039/100007786National Center for Child Health and Development (2023B-14) related to this study. She received a grant from 10.13039/501100001691JSPS KAKENHI (Grant Number 22K17791) out of submitted work. She received a lecture fee from Sato Pharmaceutical out of submitted work.

**T Fukuie** had a grant from 10.13039/100007786National Center for Child Health and Development (2023B-14) related to this study. He received lecture fees from 10.13039/100006483AbbVie, KYORIN Pharmaceutical, Maruho, Thermo Fisher Diagnostic, Torii Pharmaceutical, 10.13039/501100007132Otsuka Pharmaceutical, Viatris, and 10.13039/100004336Novartis out of submitted work. He participated on the advisory board of Torii Pharmaceutical out of submitted work. He is the member of the Committee for Japanese Guidelines for Food allergy 2021 (the Japanese Society of Pediatric Allergy and Clinical Immunology) out of submitted work.

**Y Ohya** had a grant from 10.13039/100007786National Center for Child Health and Development (2023B-14) related to this study. He received grants from 10.13039/100009619Japan Agency for Medical Research and Development (Grant Number: JP16ek0410027h0001, JP17ek0410027h0002, JP18ek0410027h0003, JP19ek0410054h001, JP20ek0410054h0002 and JP21ek0410054h0003, JP 21ek0410067h0002 and JP19ek0410037h0003), Health Labour Sciences Research Grant (Grant Number: 202111023A, 201913001B, 201911035B, and 201913007B), 10.13039/501100001691JSPS KAKENHI (Grant Number: 18H03101, 19H03569, and 20H01622), 10.13039/100007786National Center for Child Health and Development (2019E-1), Alcare, Fam's Baby, Kao, 10.13039/501100012030Yakult, Duskin, Maruho, 10.13039/100004336Novartis, Torii Pharmaceutical, and Natural Science, and consulting fees from bcase, 10.13039/100006483AbbVie, Kao, Maruho, Otsuka pharmaceutical, 10.13039/100004339Sanofi/10.13039/100009857Regeneron, Torii/Japan Tabacco, and 10.13039/100004319Pfizer out of submitted work. He received lecture fees from 10.13039/100006483AbbVie, Eli Lilly, Kyorin, Maruho, 10.13039/501100012351Mitsubishi Tanabe Pharma, Pola Pharma, 10.13039/100004339Sanofi, Shino-test, Sysmex, Shiseido, Thermo Fisher Diagnostic, Torii Pharmaceutical, Towa Pharmaceutical, 10.13039/501100007132Otsuka Pharmaceutical, and 10.13039/100004319Pfizer out of submitted work. He participated on the advisory board of Abbvie and Sanofi out of submitted work. He was the vice-chairman of the Committee for Clinical Practice Guidelines for the Management of Atopic Dermatitis 2021 (the Japanese Society of Allergology, the Japanese Dermatology Association, and the member of the Committee for Japanese Guidelines for Food allergy 2021 (the Japanese Society of Pediatric Allergy and Clinical Immunology) out of submitted work. He is a board member of the Japanese Society of Behavioral Medicine, the vice-chairman of the Japanese Society of Pediatric Dermatology, and the president of the Japan Society of Health Science for Children out of submitted work.

## References

[1] Golding M.A., Gunnarsson N.V., Middelveld R., Ahlstedt S., Protudjer J.L.P. (2021). A scoping review of the caregiver burden of pediatric food allergy. Ann Allergy Asthma Immunol.

[2] Yamamoto-Hanada K., Pak K., Saito-Abe M. (2020). Allergy and immunology in young children of Japan: the JECS cohort. World Allergy Organization Journal.

[3] Flom J.D., Sicherer S.H. (2019). Epidemiology of cow's milk allergy. Nutrients.

[4] Miyagi Y., Yamamoto-Hanada K., Ogita H. (2021). Avoidance of hen's egg based on IgE levels should Be avoided for children with hen's egg allergy. Frontiers in Pediatrics.

[5] Skripak J.M., Matsui E.C., Mudd K., Wood R.A. (2007). The natural history of IgE-mediated cow's milk allergy. J Allergy Clin Immunol.

[6] Ebisawa M., Ito K., Fujisawa T. (2020). Japanese guidelines for food allergy 2020. Allergol Int.

[7] Tang L., Yu Y., Pu X., Chen J. (2022). Oral immunotherapy for Immunoglobulin E-mediated cow's milk allergy in children: a systematic review and meta analysis. Immun Inflamm Dis.

[8] Pajno G.B., Fernandez-Rivas M., Arasi S. (2018). EAACI Guidelines on allergen immunotherapy: IgE-mediated food allergy. Allergy.

[9] Yamamoto-Hanada K., Sato M., Toyokuni K. (2023). Combination of heat-killed Lactiplantibacillus plantarum YIT 0132 (LP0132) and oral immunotherapy in cow's milk allergy: a randomised controlled trial. Benef Microbes.

[10] Takaoka Y., Yajima Y., Ito Y.M. (2020). Single-center noninferiority randomized trial on the efficacy and safety of low- and high-dose rush oral milk immunotherapy for severe milk allergy. Int Arch Allergy Immunol.

[11] Fukuie T., Nomura I., Horimukai K. (2010). Proactive treatment appears to decrease serum immunoglobulin-E levels in patients with severe atopic dermatitis. Br J Dermatol.

[12] Miyaji Y, Yamamoto-Hanada K, Yang L, Fukuie T, Narita M, Ohya Y (2023 Dec). Effectiveness and safety of low-dose oral immunotherapy protocols in paediatric milk and egg allergy. Clin Exp Allergy.

[13] Team RC (2022). https://wwwR-projectorg/.

[14] Kataoka Y. (2014). Thymus and activation-regulated chemokine as a clinical biomarker in atopic dermatitis. J Dermatol.

[15] Shoda T, Futamura M, Yang L (2016 Nov). Timing of eczema onset and risk of food allergy at 3 years of age: A hospital-based prospective birth cohort study. J Dermatol Sci.

[16] Yamamoto-Hanada K., Suzuki Y., Yang L. (2021). Persistent eczema leads to both impaired growth and food allergy: JECS birth cohort. PLoS One.

[17] Martin PE, Eckert JK, Koplin JJ (2015 Jan). Which infants with eczema are at risk of food allergy? Results from a population-based cohort. Clin Exp Allergy.

[18] Yamamoto-Hanada K, Kobayashi T, Mikami M, PACI Study Collaborators (2023 Jul). Enhanced early skin treatment for atopic dermatitis in infants reduces food allergy. J Allergy Clin Immunol.

[19] Brough H.A., Liu A.H., Sicherer S. (2015). Atopic dermatitis increases the effect of exposure to peanut antigen in dust on peanut sensitization and likely peanut allergy. J Allergy Clin Immunol.

[20] Yamamoto-Hanada K, Ohya Y (2023 Jun 14). Skin and oral intervention for food allergy prevention based on the dual allergen exposure hypothesis. Clin Exp Pediatr.

[21] Yasudo H, Yamamoto-Hanada K, Mikuriya M, Ogino F, Fukuie T, Ohya Y (2023 Oct). Association of walnut proteins in household dust with household walnut consumption and Jug r 1 sensitization. Allergol Int.

[22] Kitazawa H, Yamamoto-Hanada K, Saito-Abe M (2019 Jul). Egg antigen was more abundant than mite antigen in children’s bedding: findings of the pilot study of the Japan Environment and Children’s Study (JECS). Allergol Int.

[23] Petersen T.H., Mortz C.G., Bindslev-Jensen C., Eller E. (2018). Cow's milk allergic children—can component-resolved diagnostics predict duration and severity?. Pediatr Allergy Immunol.

[24] Morita E., Takahashi H., Niihara H. (2010). Stratum corneum TARC level is a new indicator of lesional skin inflammation in atopic dermatitis. Allergy.

