# Supplementary material for: Predictive modeling for cow's milk allergy remission by low-dose oral immunotherapy in young children
Source: World Allergy Organ J. 2024 May 17;17(5):100910. doi: 10.1016/j.waojou.2024.100910 (PMC11126529; doi:10.1016/j.waojou.2024.100910)
Supplement: Multimedia component 1 [file mmc1.docx]

Supplemental data

Table S1. Allergic reactions by accidental cow’s milk intake except OIT

|  |  | Development set | N | Validation set | N | P value |
| --- | --- | --- | --- | --- | --- | --- |
| Allergic reactions | (%) | 36(30.0) | 120 | 24(33.8) |  | 0.7 |
| Maximum Grade | (%) |  | 36 |  | 24 | 0.302 |
| Grade1 | (%) | 21(58.3) |  | 19(79.2) |  |  |
| Grade2 | (%) | 7(19.4) |  | 2(8.3) |  |  |
| Grade3 | (%) | 8(22.2) |  | 3(12.5) |  |  |
| Symptomatic organs |  |  | 36 |  | 24 |  |
| Only Skin | (%) | 14(38.9) |  | 8(33.3) |  | 1.0 |
| Respiratory | (%) | 18(50.0) |  | 15(62.5) |  | 0.324 |
| Other than respiratory | (%) | 4(11.1) |  | 1(4.2) |  | 0.653 |
| Anaphylaxis | (%) | 14(11.7) | 120 | 5(7.0) |  | 0.434 |
| Use of epinephrine | (%) | 7(5.8) | 120 | 3(4.2) |  | 0.884 |
| Emergency room visit | (%) | 19(15.8) | 120 | 8(11.3) |  | 0.509 |

OIT: oral immunotherapy

Table S2. Blood test results and trends

|  |  | Development set | N | Validation set | N | P value |
| --- | --- | --- | --- | --- | --- | --- |
| 1st visit |  |  |  |  |  |  |
| Total IgE(mean, SD) | IU/mL | 818.0±1685.0 | 118 | 696.6±1139.2 | 71 | 0.556 |
| CM sIgE (mean, SD) | UA/mL | 31.4±34.6 | 120 | 30.8±34.3 | 71 | 0.905 |
| Casein sIgE (mean, SD) | UA/mL | 27.3±34.3 | 84 | 28.0±32.7 | 55 | 0.898 |
| class |  |  | 120 |  | 71 | 0.807 |
| <=0.34 | (%) | 1(0.8) |  | 1(1.4) |  |  |
| 0.35-0.69 | (%) | 1(0.8) |  | 1(1.4) |  |  |
| 0.70-3.49 | (%) | 19(15.8) |  | 15(21.1) |  |  |
| 3.50-16.99 | (%) | 43(35.8) |  | 20(28.2) |  |  |
| 17.49.99 | (%) | 29(24.2) |  | 16(22.5) |  |  |
| 50-99.99 | (%) | 9(7.5) |  | 8(11.3) |  |  |
| 100- | (%) | 18(15) |  | 10(14.1) |  |  |
| TARC | pg/mL | 1930.8±1834.4 | 111 | 1888.1±2782.3 | 68 | 0.911 |
|  |  |  |  |  |  |  |
| Before OIT |  |  |  |  |  |  |
| Total IgE(mean, SD) | IU/mL | 689.1±1250.9 | 117 | 680.9±1121.4 | 71 | 0.963 |
| CM sIgE (mean, SD) | UA/mL | 30.2±34.1 | 120 | 29.8±33.5 | 71 | 0.936 |
| Casein sIgE (mean, SD) | UA/mL | 24.7±32.1 | 88 | 27.0±32.2 | 57 | 0.664 |
| class |  |  |  |  |  | 0.979 |
| <=0.34 | (%) | 0(0) |  | 0(0) |  |  |
| 0.35-0.69 | (%) | 3(2.5) |  | 1(1.4) |  |  |
| 0.70-3.49 | (%) | 24(20.0) |  | 15(21.1) |  |  |
| 3.50-16.99 | (%) | 39(32.5) |  | 21(29.6) |  |  |
| 17.49.99 | (%) | 23(19.2) |  | 16(22.5) |  |  |
| 50-99.99 | (%) | 16(13.3) |  | 8(11.3) |  |  |
| 100- | (%) | 15(12.5) |  | 10(14.1) |  |  |
| TARC | pg/mL | 1421.9±1647.2 | 109 | 1366.4±1225.4 | 62 | 0.803 |
|  |  |  |  |  |  |  |
| 1 year after the start of OIT |  |  |  |  |  |  |
| Total IgE(mean, SD) | IU/mL | 900.8±1341.5 | 109 | 739.1±961.7 | 67 | 0.355 |
| CM sIgE (mean, SD) | UA/mL | 27.9±35.6 | 108 | 22.6±31.0 | 65 | 0.307 |
| Casein sIgE (mean, SD) | UA/mL | 21.7±31.6 | 77 | 22.6±31.2 | 50 | 0.866 |
| Class |  |  | 109 |  | 67 | 0.0818 |
| <=0.34 | (%) | 2(1.8) |  | 3(4.5) |  |  |
| 0.35-0.69 | (%) | 8(7.3) |  | 4(6.0) |  |  |
| 0.70-3.49 | (%) | 30(27.5) |  | 15(22.4) |  |  |
| 3.50-16.99 | (%) | 24(22.0) |  | 24(35.8) |  |  |
| 17.49.99 | (%) | 21(19.3) |  | 8(11.9) |  |  |
| 50-99.99 | (%) | 9(8.3) |  | 10(14.9) |  |  |
| 100- | (%) | 15(13.8) |  | 3(4.5) |  |  |
| TARC | pg/mL | 835.9±536.8 | 106 | 857.9±594.2 | 63 | 0.81 |
| Time from start of OIT to blood test (mean, SD) | month | 12.3±4.2 | 109 | 12.1±3.0 | 67 | 0.718 |

SD: standard deviation, CM: cow’s milk, OIT: oral immunotherapy, TARC: thymus and activation-regulated chemokine

Table S3. Results of internal and external validation in the primary outcome

|  | Internal validation | External validation | |
| --- | --- | --- | --- |
|  | ROC-AUC  (95%CI) | ROC-AUC  (95%CI) | ICC  (95%CI) |
| Model 1 | 0.80  (0.72, 0.88) | 0.78  (0.67, 0.89) | 0.83  (0.47, 0.95) |
| Model 2 | 0.80  (0.72, 0.88) | 0.80  (0.69, 0.91) | 0.86  (0.55, 0.96) |
| Model 3 | 0.83  (0.76, 0.91) | 0.89  (0.80, 0.97) | 0.88  (0.62, 0.97) |

Model 1: CM- sIgE before OIT + age at beginning OIT, Model 2: Model 1 + TARC before OIT initiation, and Model 3: Model 2 + CM-sIgE 1 year after OIT

Table S4. Results of internal and external validation in the secondary outcomes

|  | Internal validation | External validation | |
| --- | --- | --- | --- |
| Results of internal and external validation in the secondary outcome (drink 10 ml in the 1st 1year) | | | |
|  | ROC-AUC  (95%CI) | ROC-AUC  (95%CI) | ICC  (95%CI) |
| Model 1 | 0.80  (0.73, 0.88) | 0.69  (0.57, 0.81) | 0.71  (0.21, 0.92) |
| Model 2 | 0.81  (0.73, 0.89) | 0.70  (0.57, 0.82) | 0.66  (0.12, 0.90) |
| Model 3 | 0.83  (0.76, 0.90) | 0.80  (0.69, 0.90) | 0.90  (0.67, 0.97) |
| Results of internal and external validation in the secondary outcome (drink 10 ml in the 1st 2year) | | | |
| Model 1 | 0.83  (0.76, 0.90) | 0.74  (0.62, 0.86) | 0.80  (0.41, 0.95) |
| Model 2 | 0.84  (0.77, 0.92) | 0.74  (0.62, 0.86) | 0.88  (0.61, 0.97) |
| Model 3 | 0.89  (0.83, 0.96) | 0.87  (0.77, 0.96) | 0.92  (0.73, 0.98) |
| Results of internal and external validation in the secondary outcome (drink 10 ml in the 1st 3year) | | | |
| Model 1 | 0.81  (0.73, 0.89) | 0.80  (0.69, 0.91) | 0.86  (0.56, 0.96) |
| Model 2 | 0.82  (0.73, 0.90) | 0.80  (0.69, 0.91) | 0.93  (0.77, 0.98) |
| Model 3 | 0.89  (0.82, 0.95) | 0.91  (0.84, 0.98) | 0.96  (0.87, 0.99) |

Model 1: CM- sIgE before OIT + age at beginning OIT, Model 2: Model 1 + TARC before OIT initiation, and Model 3: Model 2 + CM-sIgE 1 year after OIT
